# Measurement properties of external training load variables during standardised games in soccer: Implications for training and monitoring strategies

**DOI:** 10.1371/journal.pone.0262274

**Published:** 2022-01-21

**Authors:** Jo Clubb, Chris Towlson, Steve Barrett

**Affiliations:** 1 School of Sport, Exercise and Rehabilitation, University of Technology Sydney, Sydney, Australia; 2 Department of Sport, Health and Exercise Science, University of Hull, Hull, United Kingdom; 3 Sport Science, Performance Analysis, Research and Coaching (SPARC), PlayerMaker, London, United Kingdom; Universidade de Evora, PORTUGAL

## Abstract

The aim of this study was to assess the measurement properties of external training load measures across three formats of standardised training games. Eighty-eight players from two English professional soccer clubs participated in the study spanning three consecutive seasons. External training load data was collected from three types of standardised game format drills (11v11, 10v10, 7v7+6) using Global Positioning Systems. For each external training load metric in each game format, the following measurement properties were calculated; coefficient of variation (CV%) to determine between- and within-subject reliability, intraclass coefficient correlation (ICC) to determine test-retest reliability, and signal-to-noise ratio (SNR) to determine sensitivity. Total distance (TD) and PlayerLoad^™^ (PL) demonstrated *good* sensitivity (TD SNR = 1.6–4.6; PL SNR = 1.2–4.3) on a group level. However, a wide variety of within-subject reliability was demonstrated for these variables (TD CV% = 1.7–36.3%; PL CV% = 4.3–39.5%) and corresponding intensity measures calculated per minute. The percentage contribution of individual planes to PL showed the lowest between-subject CV% (CV% = 2–7%), although sensitivity varied across formats (SNR = 0.3–1.4). High speed running demonstrated *poor* reliability across all three formats of SSG (CV% = 51–103%, ICC = 0.03–0.53). Given the measurement properties of external training load measures observed in this study, specifically the within-subject variation, reliability across trials of standardised training games should be calculated on an individual level. This will allow practitioners to detect worthwhile changes across trials of standardised game format drills. Such information is important for the appropriate implementation of training and monitoring strategies in soccer.

## Introduction

Training games are used extensively in soccer training with a variety of formats, with the aim to develop players’ technical ability and tactical awareness, while concurrently targeting physiological capacities [1–3]. The inclusion and organisation of such training is considered to provide an internal training response (physiological [4,5], biomechanical [6] and psychological [4]), which practitioners attempt to capture via measures of internal load, such as heart rate or ratings of perceived exertion [4]. This response is largely governed by the accrued external training load (e.g., distances covered at different speeds [5]), which is defined as the external stimulus applied to the athlete measured independently of their internal characteristics [4]. Modifications to the constraints applied to training games have been shown to substantially influence these training processes and outcomes [1,7]. As such, practitioners must have an understanding of how these constraints may be manipulated to target technical, perceptual and physical outcomes, relative to player development and match-play performance [3].

In soccer these constraints can include the number of players, pitch size, training prescription (sets, repetitions and work: rest ratios), technical rules (limited touches, position on pitch to score), the inclusion of goalkeepers and coach encouragement [1,8,9]. Yet while such training games may aim to develop physical capacities due to the stochastic nature of this training, it may be difficult to precisely prescribe and periodise the external training load undertaken [10]. This is important, as this method of training is often employed in situations where training loads need to be well-considered (i.e., to elicit certain physiological adaptations, return to play etc.). Despite this, research has suggested that monitoring the internal and/or external training loads undertaken by players during training drills may allow for fatigue detection [11,12]. Specifically, regularly performing “standardised” versions of training games, whereby such drill constraints are kept consistent, may be used to assess fatigue status in-situ [11]. However, Rowell and colleagues [11] presented unclear findings suggesting individual variability was demonstrated in some external load metrics. Further understanding of the measurement properties of such metrics within standardised training games is required, in order to determine the potential noise that might be associated with such formats, especially if they are to be used as an assessment of fatigue status.

It is reasonable to assume that variability in training loads (within- and between-individuals) during training games may extend past constraint alternations, having implications for physical training and fatigue monitoring strategies. Yet while research has investigated some of these measurement properties, it has primarily been conducted with focus on participant numbers and specifically, smaller small sided games (SSG) (e.g. 4v4, 2v2 [1,2]), with no research to date investigating the reliability and sensitivity of commonly used medium and large sided games in soccer.

Soccer practitioners commonly monitor external training loads using micro-electromechanical systems (MEMS), such as global positioning systems (GPS) and accelerometers [13,14]. Here, metrics such as total distance covered, number of efforts above absolute and/or relative speed thresholds, maximum velocity, and acceleration/deceleration efforts across different formats are typically reported for analysis [7,15]. Caution has been advised when using MEMS devices to measure external training load during training drills due to some difficulty in capturing complex movement patterns (coupled with decreased task duration [16,17]). However, if practitioners appropriately consider the validity and reliability of devices used and metrics analysed these may be overcome [17,18]. In addition, traditional locomotor analysis fails to account for movements in multiple planes of movement such as tackles, changing direction and jumping [19,20]. Tri-axial accelerometers have been increasingly used as a measure of external training load in team sports, most commonly utilising PlayerLoad^™^ (PL) [20,21]. High levels of validity and reliability have been shown for overall PL and each of the individual planes (vertical (PL_V_), medio-lateral (PL_ML_) and anterior-posterior (PL_AP_)) between soccer matches (coefficient of variation; CV = 6.4%) and within a soccer specific simulation (Intraclass coefficient correlation; ICC = 0.80–0.99) [20]. Consequently, recommendations to use PL as a measure of external training load would allow practitioners to detect meaningful differences in accumulated load [20,21].

Despite this, limited information is available on the variability of PL during training games and the effect of different formats [7,15]. This information will likely be useful to practitioners given that it provides greater detail on the movement strategies performed by players during such games. Further, despite a body of research examining SSG [3,8,9], insufficient attention has been paid to the variability of movement patterns during such training drills [22]. Yet, external training load is often central to training prescription and return to play strategies [23,24] as well as to player support monitoring systems [25]. As such, a greater understanding of the measurement properties of this training load (dose) is required. Therefore, the aim of the current study was to examine the measurement properties of external training load measures, specifically the within- and between-subject reliability and sensitivity, with different formats of standardised training games commonly performed in soccer.

## Materials & methods

### Subjects

Eighty-eight male, elite soccer players (Age: 26.5 ± 5.8 years; stature: 1.82 ± 0.07m; body-mass: 78.8 ± 7.7kg) were recruited from two English professional teams, one in the top domestic tier (Premier League) and the other in the second tier (Championship). The study gained ethical approval study (1011137) from a university departmental ethics committee prior to the commencement of the study. As the data reported in this retrospective study was collected as part of the routine data monitoring of players in industry practice, informed consent was not deemed necessary [26].

### Procedures

Data was collected during the 2014/2015, 2015/2016 and 2016/2017 seasons from two English professional soccer teams. To provide valid and reliable information, each outfield player wore a MEMS device (Optimeye S5, Catapult Sports, Melbourne, Australia; Firmware version- 6.88–6.72), in a customized, tight-fitting neoprene garment (positioned between the scapulae) [27], as part of their daily monitoring routines within their respective training sessions at each club. These devices were taken outside and activated 15–30 minutes beforehand to attenuate erroneous data owing to poor GPS signal quality [28]. Each player wore the same unit for each session.

#### Accelerometry

The Optimeye S5 MEMS device contains a tri-axial piezoelectric linear accelerometer (Kionix: KXP94) sampling at a frequency of 100-Hz. The output of the accelerometer measures ±13g, with each device containing its own microprocessor with a 1GB flash memory and USB interface to store and download data. From this, PL was calculated by and exported from the manufacturer’s software as the sum of the instantaneous rate of change from the individual planes (PL_V_, PL_ML_, and PL_AP_) [20,21], expressed in arbitrary units (au). The percentage contribution of the individual component planes to PL were also exported from the software.

As per previous studies [11,20,29], PL was presented relative to the duration of the game (PL/min) and integrated with GPS data to calculate PL/metre. Data were recorded throughout training drills using the Catapult software (Sprint 5.1.7, Catapult Sports, Melbourne, Australia). Prior to the start of each season, units were calibrated using the manufacturers jig to ensure values were set within the manufacturers guidelines [28]. Specifically, the device was orientated and placed stationary in each plane of movement and recordings were set at 1g for that position to reduce any bias or drift [20]. Monthly checks of the calibration values were monitored to ensure the calibration values remained within the manufacturer’s calibration values throughout the testing period.

#### Time-motion analysis

The Optimeye S5 contains a 10-Hz GPS chip to record time-motion data. External load variables monitored included total distance (TD), metres per minute (m/min) and high-speed running (HSR). Commonly, HSR has been assessed via absolute and/or relative thresholds [14,30] therefore; both were included in this study. At one team, an absolute threshold (HSRa) of 5.5m/s for HSR was used [31]. At the other team, a relative threshold (HSRr) of >65% of each individual’s maximal velocity for HSR was used [32]. Each individual’s maximum velocity was determined by 10-Hz GPS data tracked across the season, as previous work has shown no significant differences exist for speed measures captured using timing gates and GPS technology [33]. Peak velocities reached by individuals were monitored daily and when a new maximum was reached, the individual’s maximum velocity was changed on the tracking system from that point onwards. For the purpose of this study, all data was then updated retrospectively with the players’ maximum velocity achieved throughout the season. As per previous methods [34], all dwell times for the variables were set to 0.2s. Data was only included if the number of satellites exceeded 6, a horizontal displacement of positioning (HDOP) was less than 1.5 and the IMF (intelligent motion filter) was switched on in the software.

#### Standardised training games

The training drills were prescribed by the respective head coaches with no intervention by sports science staff. Three different formats according to the number of players involved, standardised for all other constraints as shown in Table 1, were included in the study. At one team, an 11v11 (trials = 14; cases = 236) and 10v10 (trials = 10; cases = 432) format were performed. At the other team, a 7v7+6 (trials = 6; cases = 92) was performed. The games were consistently played on the training day prior to the next match and at least 48 hours after the previous match. Coaches were asked to maintain a consistent level of encouragement throughout, with trials excluded if any alterations were made to the games. Subjects were included if they had carried out at least three trials of the same game format.

**Table 1:** Standardised conditions of game formats.

Condition	11v11	10v10	7v7+6
Players	2 teams of 11, including a Goalkeeper on each team	2 teams of 10, including a Goalkeeper on each team	2 teams of 7, including a Goalkeeper on each team. Plus a third team of 6 outfield players on the boundary of the pitch to act as ‘wall players’.
Dimensions	60x40m	60x40m	22.9x16.5m
Sets	2 sets	2 sets	6 sets in total; 4 working sets and 2 as ‘wall players’.
Time On Per Set	10 minutes	10 minutes	2 minutes
Time Off Between Sets	2 minutes	2 minutes	45 seconds
Scheduling of Game	On the training day prior to the next match and at least 48 hours after the previous match		

### Statistical analyses

Data are presented as mean ± standard deviation (SD). Between-trial reliability of external training load variables for each game format was assessed using the percentage of coefficient of variation (CV%). This was calculated for each external load variable within each game format, using a custom spreadsheet in Microsoft Excel [35]. In order to assess the variability across trials for each player, within-subject CV% was also calculated for each external load variable across the standardised game formats. This was calculated for each individual using their between-day variation by dividing the individual’s SD by the individual’s mean and multiplying by 100. In order to demonstrate the differences and applications between commonly utilised reliability measurement properties, the authors present findings using both—between-trial and within-subject—methods.

The smallest worthwhile change (SWC) can be used to assess meaningful differences in performance [36]. The SWC was calculated as 0.2 of the between-player SD. In addition, test-retest reliability of the external training load variables were reported as the intra-class correlation coefficient (ICC) ± 90% confidence intervals (CI) using a custom spreadsheet [35]. The following criteria were used to interpret the ICC coefficients: < 0.50 poor, 0.50–0.75 moderate, 0.75–0.90 good, ≥ 0.90 excellent [37].

It is also important to consider the sensitivity of a measure, because absolute reliability does not necessarily mean a metric is sensitive to detecting a change (signal) greater than the error (noise) [38]. The signal-to-noise ratio (SNR) was calculated for each external load metric by dividing the weekly variation in a measure (% change in group mean) by the between-trial reliability (CV%) and then taking the average of all trials [38]. This was assessed using the same reliability spreadsheet [35]. The SNR was classified as good if greater than 1 and poor if less than 1 [38].

In response to calls in sports science to utilise data analysis and visualisation techniques that allow the reader to appreciate distribution and outliers, a violin plot was used to present within-subject CV% [39]. A violin plot is a combination of a box plot and a density plot. The box plot represents the median, the interquartile range and 95% confidence limits. The density plot represents the distribution shape, with a wider plot representing a higher frequency. The shape of the violin plot represents the probability density, with a higher likelihood of seeing an individual data point fall within the thicker part of the plot [39]. Outliers are shown as individual data points. By using this method to display the within-subject CV%, the reader can observe the variability in reliability of each external load variable within their playing group.

## Results

Mean external training load variables for each game format and the SWC are shown in Table 2. The 10v10 condition elicited the highest mean TD (2115.2 ± 243.7 m) in comparison to the 11v11 (2078.6 ± 250.5 m) and 7v7+6 (1106.7 ± 136.0 m). There was a similar pattern with the mean PL in the 10v10 (208.2 ± 37.2 AU) compared to the 11v11 (200.8 ± 38.1 AU) and 7v7+6 (113.8 ± 18.6 AU) formats. Differences in external load metrics across the different game formats were not statistically evaluated.

**Table 2:** Activity profile metrics and reliability for different formats of standardised soccer games.

Variable	Mean ± SD			SWC			Between-subject CV%			SNR			ICC (90% CI)		
	10v10	11v11	7v7+6	10v10	11v11	7v7+6	10v10	11v11	7v7+6	10v10	11v11	7v7+6	10v10	11v11	7v7+6
TD	2115.2 ± 243.7	2078.6 ± 250.5	1106.7 ± 136.0	48.7	50.1	27.2	6.1	6.5	7.9	2.0	1.6	4.6	0.73	0.74	0.59
										Good	Good	Good	(0.63–0.83)	(0.64–0.84)	(0.41–0.76)
MPM	99.3 ± 11.4	98.5 ± 11.9	67.8 ± 8.2	2.3	2.4	1.6	6.1	6.5	7.9	0.8	1.1	0.7	0.75	0.75	0.63
										Poor	Good	Poor	(0.65–0.84)	(0.64–0.84)	(0.45–0.78)
HSRa	67.6 ± 45.9	51.6 ± 39.1		9.2	7.8		78.9	102.5					0.49	0.53	
													(0.36–0.64)	(0.36–0.70)	
HSRr			7.3 ± 9.2			1.8			51.4						0.03
															(-0.1–0.25)
PL	208.2 ± 37.2	200.8 ± 38.1	113.8 ± 18.6	7.4	7.6	3.7	7.9	8.9	8.5	1.2	1.2	4.3	0.83	0.81	0.79
										Good	Good	Good	(0.75–0.90)	(0.73–0.89)	(0.66–0.89)
PL/min	9.8 ± 1.7	9.5 ± 1.8	7.0 ± 1.2	0.3	0.4	0.2	8.0	8.9	8.5	0.4	0.5	0.6	0.83	0.82	0.79
										Poor	Poor	Poor	(0.76–0.90)	(0.74–0.89)	(0.66–0.88)
PL/metre	0.10 ± 0.01	0.10 ± 0.01	0.10 ± 0.01	0.002	0.002	0.002	3.8	4.4	5.0	0.7	0.8	0.4	0.91	0.88	0.79
										Poor	Poor	Poor	(0.87–0.95)	(0.83–0.93)	(0.67–0.89)
PL_AP_ (%)	23.9 ± 1.5	24.5 ± 1.7	25.0 ± 2.0	0.3	0.3	0.4	3.7	4.9	6.7	0.4	1.4	0.3	0.66	0.53	0.39
										Poor	Good	Poor	(0.55–0.78)	(0.39–0.68)	(0.19–0.61)
PL_ML_ (%)	25.8 ± 1.5	25.6 ± 1.6	27.2 ± 1.4	0.3	0.3	0.3	2.9	3.2	3.4	0.7	0.8	0.3	0.79	0.76	0.59
										Poor	Poor	Poor	(0.70–0.87)	(0.66–0.85)	(0.41–0.76)
PL_V_ (%)	49.3 ± 2.1	48.8 ± 2.2	46.7 ± 1.9	0.4	0.4	0.4	2.0	2.1	2.9	0.7	1.2	0.3	0.80	0.80	0.56
										Poor	Good	Poor	(0.72–0.88)	(0.71–0.88)	(0.37–0.74)

TD—Total Distance; MPM—Metres per minute; HSRa—High Speed Running (Absolute) distance above 5.5m/s; HSRr—High Speed Running (Relative) distance covered above 65% individual maximum velocity; PL—PlayerLoad^™^ Vector Magnitude; PL/min—PlayerLoad^™^ per minute; PL/metre—PlayerLoad^™^ per metre; PL_AP_ (%)—% contribution to PlayerLoad^™^ in the Anterior-Posterior plane; PL_ML_ (%)—% contribution to PlayerLoad^™^ in the Medial-Lateral plane; PL_V_ (%)—% contribution to PlayerLoad^™^ in the Vertical plane; SD—Standard deviation; SWC—Smallest Worthwhile Change; CV%—Coefficient of variation; SNR—Signal-to-noise ratio, average of weekly % change in group mean divided by CV%; ICC—Intraclass correlation coefficient; CI—Confidence intervals.

The between-subject CV, SNR, and ICC of the external load metrics are presented in Table 2. Data for TD demonstrated good (1.6 to 4.6) SNR in all three formats. Reliability assessed via the ICC was moderate-to-good for the 10v10 (0.73 to 0.83) and 11v11 (0.64 to 0.84). However, values ranged from poor-to-good (0.41 to 0.76) for the 7v7+6. That said, relative distance expressed as m/min for the 7v7+6 showed moderate-to-good (0.45 to 0.78) reliability. However, the 10v10 and 7v7+6 formats displayed poor sensitivity (0.7 to 0.8) for relative distance. Activities performed above both absolute and relative HSR thresholds demonstrated poor reliability (between-subject CV% = 51–103%; ICC = 0.03–0.53; within-subject CV% = 19–244%) across all three game formats. Given the poor reliability results, the SNR analysis for both HSR measures returned errors so this data is omitted from Table 2.

The PL, PL/min and PL/metre data demonstrated between-subject CV% below 10% for all three formats. The ICC ranged from moderate-to-excellent (0.79 to 0.91). However, when PL was calculated per minute or metre, the SNR was poor (0.4 to 0.8). The percentage contribution of the individual planes to PL demonstrated the lowest between-subject CV% (2.0 to 6.7%). However, the ICC ranged from poor to good, with a lower correlation for each respective metric in the 7v7+6 compared to the 10v10 and 11v11 drills. In the 11v11 format, PL_AP_ (%) and PL_V_ (%) showed good sensitivity (1.2 to 1.4). Across all the external load metrics, the ICC was lower in the 7v7+6 format than 10v10 and 11v11.

**Fig 1:**
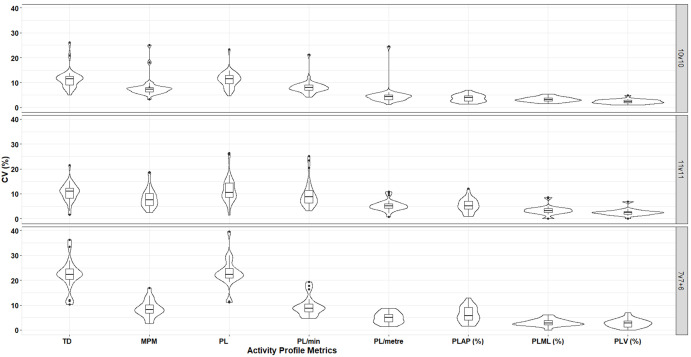
A violin plot of the within-subject CV for each external training load measure across three different SSG formats. TD—Total distance; m/min—metres per minute; PL—PlayerLoad^™^; PL/min—PlayerLoad^™^ per minute; PL/metre—PlayerLoad^™^ per metre; PL_AP_ (%)—% contribution to PlayerLoad^™^ in the Anterior-Posterior plane; PL_ML_ (%)—% contribution to PlayerLoad^™^ in the Medial-Lateral plane; PL_V_ (%)—% contribution to PlayerLoad^™^ in the Vertical plane; CV%—Coefficient of variation.

Within-subject CV% are displayed in the violin plot in Fig 1. Fig 1 highlights the existence of outliers that account for the large range of within-subject variation, such as PL and PL/min across all game formats. The figure also indicates the higher density of data around the mean in the percent contribution of the individual planes to PL, representing lower within-subject variation in these metrics. HSR metrics were excluded from this figure due to the poor reliability observed.

## Discussion

Many research studies have explored the effects of different training game formats on external training load (1, 7, 9]. In order to assess when changes in external load variables across such training games are meaningful, practitioners require an understanding of the measurement properties associated with such metrics in such settings. Recently, a greater emphasis has been placed on using such drills to drive fatigue monitoring and training strategies [11,12] and therefore, understanding group and individual movement variance is critical. The current study aimed to examine the measurement properties of external training load variables across three different training game formats. The main findings of the present study are four-fold: 1) cumulative variables of TD and PL demonstrated moderate-to-good reliability and SNR, suggesting these metrics may be sensitive to track group changes across trials. 2) Conversely, m/min, PL/min and PL/metre may not be sensitive to track changes in intensity as the noise was greater than the signal (SNR<1, except for m/min in the 11v11 format). 3) Both absolute and relative HSR showed poor reliability across the three game formats. 4) The percentage contribution of individual component planes to PL demonstrated high variation, with poor-to-good reliability according to the ICC across all formats. The range of within-subject reliability across all other variables highlights the need to consider reliability on an individual athlete level in the applied environment.

Understanding the reliability of a given variable will help sport scientists and practitioners to calculate a meaningful difference within that variable. Across different training game formats, variables such as TD, m/min, and HSR activities have previously been assessed for reliability [9,40]. Within the current study, TD and m/min demonstrated *good* between-subject CV% between 6 and 8% across all SSG formats. This is similar to previous findings that showed *good* reliability in total distance covered in 1v1, 2v2 (CV% = 6.1–7.9%; [9]) and 6v6 games (CV% < 5%; [40]). Therefore, given this level of reliability, practitioners can utilise changes in TD and m/min to calculate meaningful differences across trials in these formats of standardised games. In contrast, HSRa and HSRr showed poor reliability across the three formats, similar to previous research examining other game formats (2v2, 4v4, 6v6) [15]. While the reliability of HSRa in 6v6 games was enhanced (CV% = 12–17%) compared to the present study, the authors still concluded this was too variable to track changes in individual players [40]. Our findings relating to HSR are somewhat unsurprising given the high variability demonstrated in competitive games across a season in professional soccer [41]. It has been proposed that the individualisation of HSR thresholds according to individual fitness characteristics may provide more stability in capturing running performance [41,42]. The current findings, however, demonstrate measures of HSRr provided no improved reliability during SSGs than HSRa distance. This finding is supported by Scott and Lovell [43] who found the individualisation of speed thresholds, using both maximal sprint speed and maximal aerobic speed, did not enhance the dose-response measurement of internal training load. Taken together, these findings question the efficacy of practitioners dedicating the additional time and resources (i.e., specialist laboratory and field-based testing) necessary to establish individualised speed thresholds.

Speed and distance metrics, such as HSR, have been suggested to neglect the energetically taxing changes in velocity in different planes of motion associated within soccer activities [20]. Tri-axial accelerometer-derived variables measuring movement in three planes, such as PL, have been observed as an alternative to monitor intermittent multi-directional activities such as soccer [19,20]. Within the current study, PL had a SNR >1, suggesting that it can be used to detect meaningful differences within a specific group (i.e., team) of players. However, this was not the case when PL was made relative to time (PL/min) or distance covered (PL/metre). On an individual level, there was lower within-subject variation across PL and PL/min. Some of this variability may be extenuated to the placement of the device (between the scapulae), with suggestions that foot-mounted inertial sensors may be a more appropriate method to capture these specific movements [44]. Still, PL has been linked to alterations in acute fatigue within a fixed soccer simulation, postulating this may be able to detect alterations within an individual’s locomotor efficiency [29]. Links have been made between PL/min and the neuromuscular fatigue levels of Australian Football players [45]. Furthermore, Rowell and colleagues demonstrated reductions in PL/min during a standardised training game were associated with the same reductions in subsequent match external load metrics as those measured when countermovement jump performance (flight time: contact time) was reduced [11]. Given that such reductions had the same implications for match exercise intensity, the authors of that study postulated that PL/min could be used to assess fatigue in situ via a standardised training game, without the burden of additional jump testing [11]. Our findings, however, suggest that caution may be needed when monitoring certain PL variables during training games, due to the higher within- and between-subject variability witnessed. Nevertheless, it appears the accumulation of the PL may be highly individualised and further work is warranted to investigate if such variability is driven by fatigue and subsequent changes to movement patterns.

In addition to prior findings linking PL and PL/min to markers of fatigue, changes to the within-match contribution of individual planes of PL have been shown in both soccer match play and a simulation as a possible means for assessing fatigue in-situ [11,20,29]. In the current study, low CV%, both within- and between-subjects, were observed for the percentage contribution of individual component planes to PL. Reductions in PL_V_ (%) have been shown during elite Australian Football matches when the players started the game with existing neuromuscular fatigue [19]. However, individual variability (unclear results) was demonstrated in PL_V_ (%) during soccer match play in those experiencing fatigue [45]. In addition, associations have been made between the increased contribution of PL_ML_ and the level of neuromuscular fatigue, assessed via both a jump test and a standardised training game [11]. Evidence of fatigue-induced changes in the relative contributions of the individual component planes to the vector magnitude is of interest given the low variability of these metrics demonstrated in this study. It would therefore seem pertinent to investigate the potential association between changes in fatigue-induced movement strategies and sensitivity of the individual component planes to detect these within training games in future work.

Understanding the meaningful difference of external training load measures within an individual’s response to a given activity can help practitioners with planning and intervention strategies for the individual (e.g., training loads, recovery interventions) [11,20,38]. Taken together, it is apparent that global measures of ‘volume’ (e.g. TD, PL) present more stable measures of external training load to monitor during training games between individual players. These values however may be of less interest to practitioners than relative (per minute and metre) and higher-intensity metrics, due to the use of the latter in fatigue assessments [11], return to play [23] and physical conditioning [24] strategies. Based on the findings of the current study, we suggest caution is taken when using such volume and intensity metrics to plan and monitor external training loads; instead, practitioners may consider implementing other controlled training and assessment tools alongside standardised training drills, particularly when fatigue monitoring is of focus. Additionally, practitioners may look to use ‘live’ monitoring and feedback to communicate an individual’s actual external load compared to that which was planned, when using drills as part of conditioning and rehabilitation programming to appropriate track and, where suitable, adjust individual training loads.

The current study demonstrates a process that practitioners can undertake to better understand the variation in external training load measures across trials of a standardised training game in their own setting. Findings of the current study highlights the individual variation within a team sport setting that should be considered and assessed, especially if games are prescribed with specific external training load objectives. Some limitations of this study should also be noted. Although incorporating two teams increased the data available to analyse, the potential effect of different playing level (first and second tier) on the results is unknown. The subjects were professional male footballers and so these findings may not be replicated in other populations, such as female footballers or youth players. The training drill formats used were based on those used in each applied setting, but many other designs are frequently used (i.e., number of players, pitch size, drill rules etc.). In addition, data pertaining to prior training load and fatigue status were not collected in the current study. Future work should explore what drives this variability, how these variables are influenced by prior training load and/or fatigue, and whether these metrics are sensitive to changes in movement patterns, to provide greater insight into their capacity to be used within testing and training strategies.

## Conclusions

This study suggests the percentage contribution of the individual planes to PL demonstrated good reliability, both within- and between-subjects. Good reliability was observed for TD, m/min, PL, and PL/min on a group level but high within-subject variation was demonstrated for these variables. Therefore, it is recommended to monitor these external training load variables on an individual level. Both HSRa and HSRr showed poor reliability.

These findings suggest that practitioners can use TD, m/min, PL, and PL/min to detect meaningful group differences in the external load of standardised training games using the current formats. However, HSR may not be an appropriate metric for detecting meaningful changes. The variability in the external training load measures observed in this study demonstrates the need for greater attention on physical outcomes when prescribing games in soccer training. These findings also question the transferability of some of the research into training games (including small, medium, and large sided games) variation into the applied environment if only between-subject observations have been made. Awareness of this variability, along with monitoring of such metrics on an individual level, may assist practitioners with a better understanding of the external loads of training games in the applied environment.
